# A systematic review of factors that affect uptake of community-based health insurance in low-income and middle-income countries

**DOI:** 10.1186/s12913-015-1179-3

**Published:** 2015-12-08

**Authors:** Esther F. Adebayo, Olalekan A. Uthman, Charles S. Wiysonge, Erin A. Stern, Kim T. Lamont, John E. Ataguba

**Affiliations:** Centre for Evidence-based Health Care, Faculty of Medicine and Health Sciences, Stellenbosch University, Cape Town, South Africa; School of Public Health and Family Medicine, University of Cape Town, Observatory, South Africa; Warwick-Centre for Applied Health Research and Delivery (WCAHRD), Division of Health Sciences, Warwick Medical School, The university of Warwick, Coventry, CV4 7AL UK; Liverpool School of Tropical Medicine, International Health Group, Liverpool, Merseyside UK; Cochrane South Africa, South African Medical Research Council, Cape Town, South Africa; Women’s Health Research Unit, School of Public Health, Faculty of Health Sciences, University of Cape Town, Cape Town, South Africa; Soweto Cardiovascular Research Unit, University of the Witwatersrand, Johannesburg, South Africa; Health Economics Unit, School of Public Health and Family Medicine, University of Cape Town, Observatory, South Africa

## Abstract

**Background:**

Low-income and middle-income countries (LMICs) have difficulties achieving universal financial protection, which is primordial for universal health coverage. A promising avenue to provide universal financial protection for the informal sector and the rural populace is community-based health insurance (CBHI). We systematically assessed and synthesised factors associated with CBHI enrolment in LMICs.

**Methods:**

We searched PubMed, Scopus, ERIC, PsychInfo, Africa-Wide Information, Academic Search Premier, Business Source Premier, WHOLIS, CINAHL, Cochrane Library, conference proceedings, and reference lists for eligible studies available by 31 October 2013; regardless of publication status. We included both quantitative and qualitative studies in the review.

**Results:**

Both quantitative and qualitative studies demonstrated low levels of income and lack of financial resources as major factors affecting enrolment. Also, poor healthcare quality (including stock-outs of drugs and medical supplies, poor healthcare worker attitudes, and long waiting times) was found to be associated with low CBHI coverage. Trust in both the CBHI scheme and healthcare providers were also found to affect enrolment. Educational attainment (less educated are willing to pay less than highly educated), sex (men are willing to pay more than women), age (younger are willing to pay more than older individuals), and household size (larger households are willing to pay more than households with fewer members) also influenced CBHI enrolment.

**Conclusion:**

In LMICs, while CBHI schemes may be helpful in the short term to address the issue of improving the rural population and informal workers’ access to health services, they still face challenges. Lack of funds, poor quality of care, and lack of trust are major reasons for low CBHI coverage in LMICs. If CBHI schemes are to serve as a means to providing access to health services, at least in the short term, then attention should be paid to the issues that militate against their success.

**Electronic supplementary material:**

The online version of this article (doi:10.1186/s12913-015-1179-3) contains supplementary material, which is available to authorized users.

## Background

Many low-income and middle-income countries (LMICs) are faced with the challenge of raising sufficient funds to finance health services in an equitable way [1]. Although it is expected that governments should play a leading role in this regard, most governments in these countries are constrained by the high proportion of informal workers. Also, other economic contexts such as high public debt and population growth rate in most of these countries have made it difficult to increase government spending on health [2]. As a result only a small fraction of government revenue is allocated to providing healthcare services for the population. Similarly, the burdens of disease in these countries are higher than those in high-income countries [3]. In fact, LMICs account for 90 % of the global burden of disease and only 12 % of global health spending [3].

In many LMICs direct out-of-pocket payments dominate healthcare financing [4]. Such direct payments are inequitable and inefficient in financing healthcare services [5]. This is because they are generally regressive; accounting for a higher proportion of poorer households’ income compared to richer households [1]. Thus, many households in LMICs lack adequate financial protection; households face financial catastrophe and impoverishing effects of paying for health services out-of-pocket [6]. In fact, annual estimates show that about 44 million households (representing more than 150 million individuals) face catastrophic expenditure globally while about 25 million households (representing more than 100 million people) are impoverished because of direct healthcare payments. Over 90 % of these occur in LMICs [7].

In response to adverse effects of direct out-of-pocket payments, the World Health Organization (WHO) is encouraging countries to move towards universal health coverage (UHC). This means that everyone should have access to needed healthcare services that are effective and of acceptable quality, and no one should risk financial ruins as a result of this. This is corroborated by evidence from many LMICs showing that health sector reforms in the form of adequate insurance or prepayment schemes contribute to increasing financial protection [5, 8]. One form of such prepayment schemes that is commonly advocated for informal workers and those in rural communities is community-based health insurance (CBHI) schemes or *mutuelles de santé* in francophone African countries.

CBHI schemes are noted for the principal role of a community’s involvement in raising, pooling, allocating, purchasing and supervision of the health financing arrangement. Some of these schemes cover similar geographical entities, professional affiliations and some other joint activity. Their beneficiaries are individuals with no form of financial protection or ability to cover the cost of healthcare services; and the schemes are voluntary in nature [9]. Although CBHI schemes are criticised for the limited extent of resource generation and pooling, they have been shown to facilitate and improve access to healthcare services, especially among children and pregnant women [10, 11]. Moreover, CBHI also addresses, to some extent, healthcare challenges faced specifically by the rural poor and informal workers. However, enrolment to CBHI schemes remains a challenge mainly because of their voluntary nature [12]. In Africa only 2 million people out of an estimated population of 900 million people are enrolled in a CBHI scheme. This amounts to just 0.2 % of the catchment population [13].

Only a few studies have assessed the impact of these schemes on selected health indicators. Over the past decade, a couple of systematic reviews that assessed the impact of the CBHI schemes on health status, the use of health services and financial protection have reported mixed results [13–16]. In some case, these schemes provide some form of reductions in out-of-pocket payments [13] while in other cases there is no significant impact on out-of-pocket payments, the use of health services or health status [16]. While these systematic reviews focus on the impact of CBHI, there is a paucity of research which systematically explores the reasons for the poor enrolment [11]. We are not aware of previous systematic reviews that have summarised factors associated with uptake of CBHI. This is one of the motivations for this systematic review of the factors that affect enrolment into CBHI schemes. The review also describes the quality of existing literature and discusses the policy implications of currently available evidence.

## Methods

### Protocol and registration

The review rationale and methods were specified in advance, documented and published in a systematic review protocol [17].

### Search strategy

An exhaustive and comprehensive search was performed with the help of an information specialist, to help recognise all relevant studies in English available regardless of publication (published, unpublished, in progress or in press) status.

We searched the following electronic databases: PubMed, Cumulative Index to Nursing and Allied Health Literature (CINAHL), Scopus, Web of Science, Education Resources Information Centre (ERIC), PsycINFO, Humanities international, International Bibliography of the Social Sciences (IBSS), Sociological abstracts, Social online, Cochrane Database of Systematic Reviews (CDSR), WHO library databases (WHOLIS), Africa Index Medicus, Latin American and Caribbean Health Sciences Literature (LILACS), IndMed, Academic One file, Africa Wide, Business source premier and Journal storage (JSTOR). We used both text words and medical subject heading (MeSH). Additional file 1: Table S1 shows detailed information on the search for the PubMed database. We searched other websites including the National Bureau of Economic Research (http://www.nber.org/), Institute of Development Studies (http://www.ids.ac.uk/), International Health Economics Association (https://www.healtheconomics.org/), Canadian Institute of health Information (http://www.cihi.ca/CIHI-ext-portal/internet/EN/Home/home/cihi000001), and EconPapers (http://econpapers.repec.org/). We also checked the reference lists of all full text articles included in the review and searched grey literatures.

### Inclusion and exclusion criteria

We included all studies (controlled before-and-after studies, interrupted time series designs, cohort studies, case–control studies, cross-sectional surveys, and qualitative) that reported factors that affect the uptake of CBHI in LMICs (as defined by the World Bank).

For this review, CBHI was defined as the application of the principles of insurance by a defined community bearing in mind the cultural and social context, which is directed by a community’s choice and based on their arrangement and structures. Mutual health organisations, community health funds, rural health insurance, micro insurance, revolving drug funds and community based prepayment scheme were all considered as synonyms. To be included, the studies had to report at least one of the following primary and secondary outcomes. The primary outcomes of interest for this review were uptake of, or willingness to pay for CBHI schemes (as defined by the authors of the primary studies). The secondary outcomes included acceptability of insurance schemes, availability of health services, ability to pay, financial protection, fairness in financial contribution, and utilisation of health services.

### Study selection

Two authors (EA and KL) working independently applied the inclusion criteria to citations identified via the searches; compared their results and resolved any discrepancy by discussion and consensus. If a decision was not reached, a third author (CW) was consulted. For each identified study that met the inclusion criteria, details on study design, study population characteristics, outcome measures, and study quality were extracted.

### Quality assessment

To assess the quality of studies included, a tool was modified from the Strengthening the Reporting of Observational Studies in Epidemiology (STROBE) guidelines [18]. The risk of bias was assessed by scoring low risk = 2, moderate risk = 1, unclear = 0, high risk = minus 2. The total score was used as the summary assessment for the risk of bias. The evaluation for each study was assessed by two authors (EA and KL). In case of any discrepancy in the assessment of a study between the authors, a final decision was taken by consensus. In summary, all the studies included in this review were of strong quality, with low to moderate risk.

### Data extraction

Two authors (EA and KL) independently extracted data for each included article using a standardised data collection form. For each study, the following information was extracted: citation, study design and methodology, geographic setting, nature of CBHI, outcomes, types of analysis performed, and findings. The two authors compared the extracted data and resolved discrepancies by discussion and consensus; failing which a third author arbitrated.

### Dealing with missing data

In cases of missing or incomplete information presented in included studies, we attempted to contact authors for further information. Although we could not get contact details for some authors, none of those we contacted provided us with follow up information.

### Data synthesis

It was not possible to combine all results using meta-analyses because the included studies differed significantly in study settings, design, and outcome measures. Thus, we used a narrative synthesis; to present details for each study and discuss them in turn.

## Results

### Study selection

**Fig. 1:**
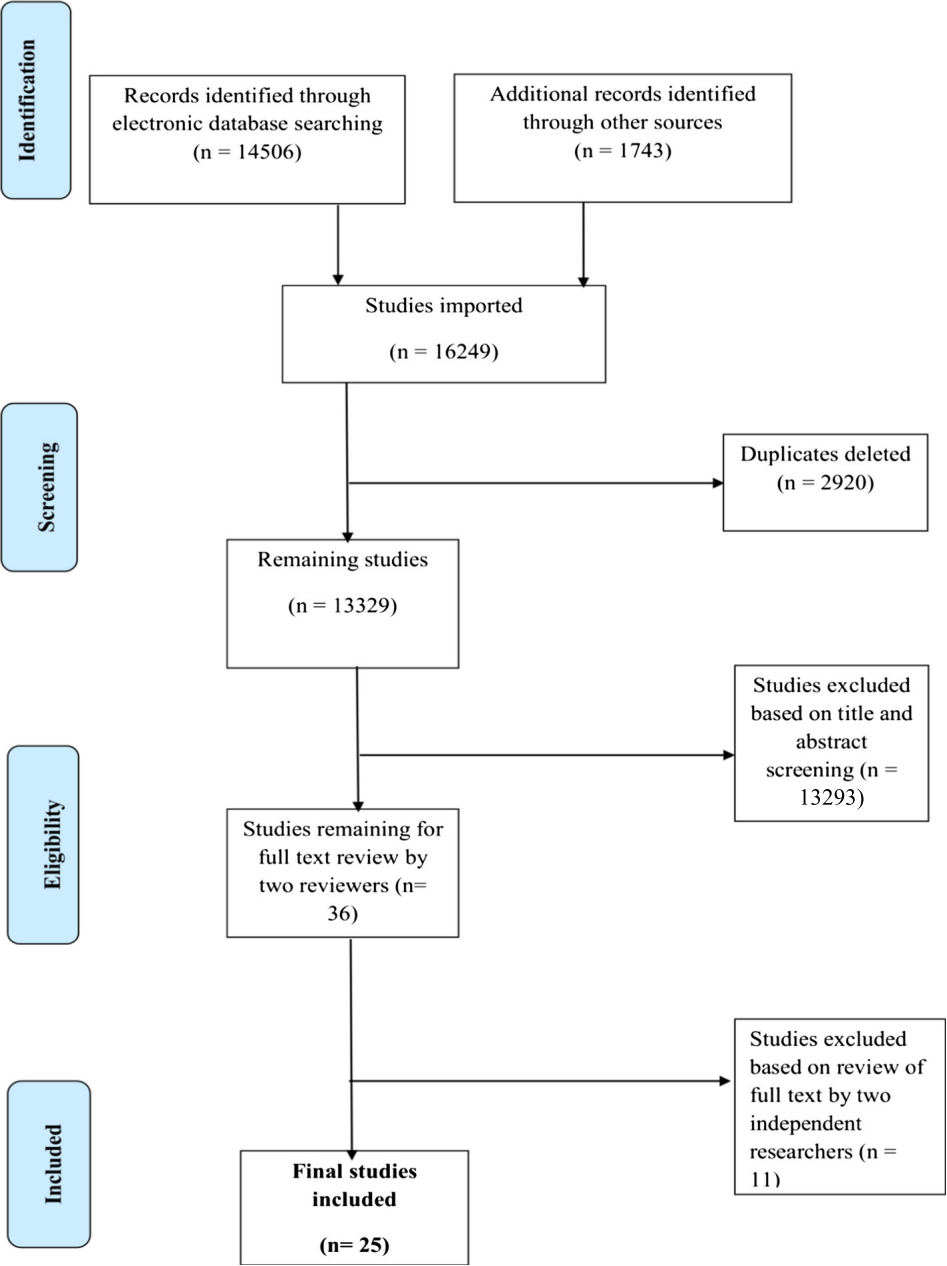
Flow chart showing the results of search and selection of studies

The process and results of study identification are outlined in a flow diagram (Fig. 1). A total of 14,506 records were identified through a comprehensive search of the electronic databases and 1743 from other sources; hence a total 16,249 records were identified in total of which 2920 were duplicates. The remaining 13,329 studies’ (after removing the duplicates) titles and abstract were screened; we excluded 13,293 clearly irrelevant records. The remaining 36 full texts were reviewed for eligibility. Among the potentially eligible publications, 11 were excluded with reasons while 25 studies were eligible for this review. We provided reasons for excluding each publication in Additional file 2: Table S2. All included studies except one were cross-sectional studies (specifically household surveys). One of the included studies used a mixed method; this was presented as a separate entry in both qualitative and quantitative studies.

### Study characteristics

A total of 25 studies were included, 18 quantitative studies, six qualitative and one mixed method. Table 1 provides a detailed summary of interventions and study results.

**Table 1:** Characteristics of studies that met inclusion criteria

Study ID (Year)	Study design	Study setting	Study outcome	Funding source
Quantitative studies				
Ataguba 2008 [39]	Cross-sectional study	Nigeria Rural setting	Willingness to pay	AusAID, IDRC, CIDA, SIDA.
Ataguba 2008 [29]	Cross-sectional study	Nigeria Rural setting	Willingness to pay	AusAID, IDRC, CIDA), SIDA.
Banwat [19]	Cross-sectional study	Nigeria Rural setting	Willingness to pay	Not reported
Binnendijk 2013 [20]	Cross-sectional study 2009–2010	India	Willingness to pay	NOW and German Federal Ministry for Economic Cooperation and Development.
Chankova 2008 [21]	Cross-country study 2004	Ghana (rural district), Mali (both rural and urban district) and Senegal (rural)	Uptake of community based health insurance	USAID
Donfouet 2011 [23]	Cross-sectional study November 2009	Cameroon Rural	Willingness to pay	ILO, African Doctoral Dissertation Research Fellowship offered by the APHRC in partnership with the IDRC
Donfouet 2013 [22]	Cross-sectional study (household survey)	Cameroon Rural	Willingness to pay	ILO, African Doctoral Dissertation Research Fellowship offered by the APHRC in partnership with the IDRC
Dong 2003 [26]	Cross-sectional study (household survey)	Burkina Faso Rural	Willingness to pay	Germany Research Foundation
Dong 2004 [25]	Cross-sectional study (household survey)	Burkina Faso	Willingness to pay	Germany Research Foundation
Dong 2009 [31]	Cross-sectional study May 2006	Burkina Faso Rural	Willingness to pay	German Research Society
Dror 2007 [27]	Cross-sectional study (household survey)	India Rural	Willingness to pay	ECCP and GTZ
Dong 2003 [24]	Cross-sectional study (household survey)	Burkina Faso Rural	Willingness to pay	German Research Society
Mathiyazhagan 1998 [28]	Survey research and heuristic/documentary research	India Rural setting	Willingness to pay	Not reported
Onwujekwe 2009 [30]	Cross-sectional study (household survey)	Nigeria Urban, semi-urban and rural area.	Willingness to pay	AFRO, Brazzaville
Oriakhi 2012 [36]	Cross-sectional study (household survey)	Nigeria Rural	Willingness to pay	Not reported
Allegri 2006 [32]	Population-based case-control study 2004	Burkina Faso	Uptake of CBHI	German Research Society
Shafie 2013 [33]	Cross-sectional study 2009	Malaysia	Willingness to pay	Universiti Sains Malaysia Short Term Grant
Ozawa 2009 [40]	Mixed method	Cambodia	Uptake of CBHI	Not reported
Binam 2004 [35]	Cross-sectional	Cameroon	Willingness to pay	Not reported
Qualitative studies				
De Allegri 2006 [41]	Semi-structured interview May–June 2004	Burkina Faso Rural	Uptake of scheme	German Research Society
Basaza 2007 [37]	Case study evaluation (semi-structure interview) November 2004–December 2005	Uganda Rural	Uptake of scheme	Ministry of Health Uganda, the DGIC Belgium and Institute of Tropical Medicine in Antwerp
Allegri 2005 [43]	In-depth interviews and semi-structured interviews May–June 2004	Rural and urban	Uptake of scheme	German Research Society
Basaza 2008 [38]	Focus group discussion October 2005–March 2006	Uganda Rural	Uptake of scheme	Not reported
Criel 2007 [42]	Focus group March 2000	Guinea-Conakry Rural	Uptake of scheme	German bilateral co-operation and the Institute of Tropical Medicine in Antwerp
Ozawa 2009 [40]	Focus group	Cambodia Rural	Uptake of scheme	UK Department for International Development
Schneider 2005 [44]	Focus group August 2000	Rwanda Rural	Uptake of scheme	Not reported.

*NOW* Netherlands Organisation for Scientific Research, *USAID* United States Agency for International Development, *APHRC* African Population and Health Research Center, *ILO* International Labour Organization, *ECCP* European Union within the EU-India Economic Cross Cultural Programme, *GTZ* Deutsche Gesellschaft für Technische Zusammenarbeit, *AFRO* African Regional Office of the World Health Organization, *IDRC* International Development Research Centre, *AusAID* Australian Agency for International Development, *IDRC* International Development Research Centre, *CIDA* Canadian International Development Agency, *SIDA* Swedish International Cooperation and Development Agency

### Socio-demographic factors influencing the uptake of CBHI

**Fig. 2:**
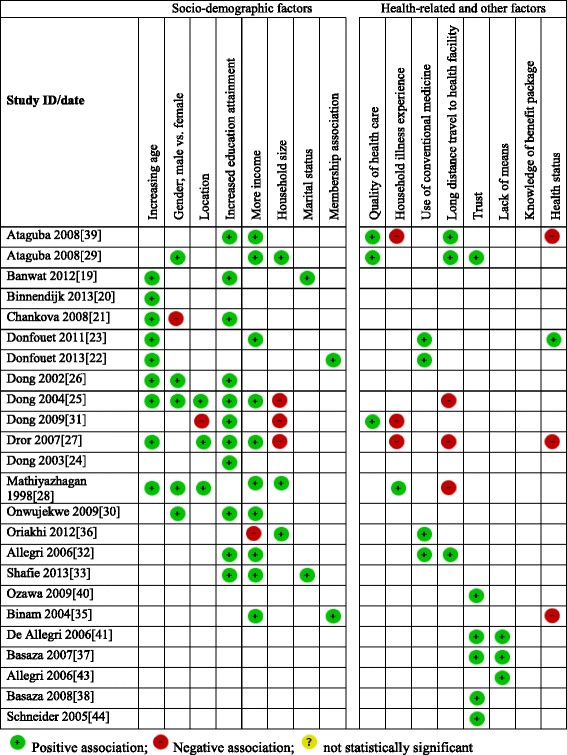
Summary results from included studies

Summary results for socio-demographic factors reported in the included studies are summarised in Fig. 2 and Additional file 3: Table S3. Age of the participants has a statistically significant association with the uptake of the scheme, and studies conducted in Nigeria, India, Ghana, Mali, Senegal, Cameroon and Burkina Faso have revealed that young individuals (between ages 30 and 49) were more willing to pay [19–28] as compared to the older individuals. At the household level, older age of household head was positively associated with enrolment in Ghana, Mali and Senegal [21]. In terms of gender, male headed households in Burkina Faso and Nigeria were found to be more likely to enrol as compared to female headed households [25, 29, 30] and at the individual level, men were found to be willing to pay more for CBHI than women in Burkina Faso, Nigeria and India [25, 26, 28, 30]. This differs from the results of studies conducted in Ghana, Mali and Senegal [21], which revealed that female-headed households were more likely to enrol in CBHI schemes.

Geographic location (rural or urban) also affected enrolment. Some studies conducted in Burkina Faso and India showed that urban dwellers were willing to pay less as compared with rural dwellers [25, 27, 28] while the opposite was recorded in another study conducted in Burkina Faso [31]. Education also played a key role in uptake of CBHI, as all studies conducted in Nigeria, Ghana, Mali, Senegal, Burkina Faso, India and Malaysia that reported this variable found that the less educated were willing to pay less compared to the more educated [19, 21, 24–27, 30–33] at both household and individual levels. The studies measured willingness to pay rather than the ability to pay, although the former can be used as proxy to measure the latter.

Wealthier households and individuals (richest quintile or as defined by the study) were more willing and able to pay more for health insurance than the less wealthy as seen in studies carried out in Cameroon, Burkina Faso, India, Nigeria and Malaysia [23, 25, 27–30, 32–35]. However one study conducted in Nigeria reported differently in terms of wealth quintile and enrolment whereby those with high income were less likely to pay than those with lower income [36]. Findings from qualitative studies also show that wealth quintile was stated as a socio-demographic factor revolving around the uptake of the scheme, and as shown by quantitative studies, affordability is a key factor affecting enrolment. Non-enrolled individuals collectively identified a lack of financial means as the primary reason for not enrolling in Burkina Faso and Uganda [32, 37, 38] (Additional file 3: Table S3).

In addition, household size was another key factor that was found to affect uptake of CBHI schemes. Studies conducted in India and Nigeria found that larger households (six members and above) were willing to pay higher amounts than relatively smaller households [28, 29, 36]. This differs from what was reported in some other studies conducted in Burkina Faso and India [25, 27, 31]. Where larger households dropped out of the scheme, this was likely as a result of the huge financial burden faced by households when they seek health care. Some studies carried out in Nigeria and Malaysia associated marital status to the uptake of the scheme. Single individuals were more willing to pay than married couples [19, 33]. Households that were members of an existing association in the community were more willing to enrol into the scheme as seen in Cameroon [22, 35], which reveals the role of solidarity and social cohesion on willingness to pay for the scheme.

### Health related factors influencing uptake of CBHI

Summary results for health related factors influencing the uptake of CBHI are presented in Fig. 2 and Additional file 2: Table S2. The quality of health care is another key factor that was found to influence the uptake of the scheme. Individuals or households that perceived quality of care as good were found to be more willing to pay than those who perceived the quality with less admiration as reported in Burkina Faso and Nigeria [31, 39]. One study conducted in Nigeria linked the quality of health care and distance together in the sense that, households that perceive quality of health care centres close to them as poor are willing to enrol into the scheme and are willing to pay higher [29]. This would enable them have access to other facilities that are far away but with good quality.

In addition, household illness experiences were also found to determine enrolment, and the results of some included studies carried out in Burkina Faso showed that households that have recorded sick members are less willing to pay than their counterparts [34]. It is cognisant to note that no particular illness was stated in any of the studies. Another empirical study conducted in India reported that households with more sick members were willing to pay more [28], which supports the notion that families with high illness rates or more prone to being ill, have a greater tendency to participate or to be members of the health prepayment scheme. Alternatively, lower number of illness episodes in a specified period of time led to higher drop-out from the scheme as seen in India and Burkina Faso [27, 31]. Health status also determined enrolment as seen in India, Cameroon and Nigeria [27, 35, 39] as individuals with better health status were willing to pay less amounts for health insurance compared with individuals with poorer health status [23].

The use of modern medicine is also an important factor for enrolling into CBHI since the scheme requires the regular use of modern means of treatment; hence those who use modern medicine have been found to be willing to pay more than those who use other means of treatment as revealed by studies conducted in Cameroon, Burkina Faso and Nigeria [22, 23, 32, 36]. Trust in CBHI was also reported to affect willingness to pay in Nigeria and Cambodia, as household heads that have greater trust in the scheme were willing to pay higher amounts than their counterparts [29, 40]. Trust was also stressed in almost all qualitative studies conducted in Uganda, Cambodia and Burkina Faso [37, 38, 40, 41].

One other factor that affected enrolment is household travel distance (distance was not qualified in the three studies that considered it as a factor that affect enrolment) to access health care. Households in Nigeria and Burkina Faso travelling longer distances were found to be more willing to pay for CBHI than those that needed to travel less distance [29, 32, 39]. This result diverges from some other studies conducted in Burkina Faso and India that reported the opposite association between distance required to access healthcare and willingness to pay, whereby fewer people were enrolled [25, 27, 28]. However, long distance to health facilities was not explicitly defined in these studies; hence a general pattern was not defined across the studies. In addition, perception of the quality of health care was also found to affect enrolments. Respondents criticised excessive prescribing, long waiting times, differential treatment, health provider’s attitude and technical incompetence amongst providers, irrespective of enrolment status [41–44] as issues that affect uptake of CBHI schemes in Burkina Faso, Guinea-Conakry and Rwanda. Poor knowledge of the benefit package and poor understanding of the notion of the scheme was also found to affect enrolment in Uganda and Burkina-Faso [37, 38, 41]. Institutional rigidities in payment modality and timing of the enrolment campaign in relation to seasonal revenue fluctuations [43] were also found to contribute to the uptake of CBHI in Burkina-Faso. Low-level community participation and involvement in the decision-making process [37, 38] and lack of “solidarity acts in a community” hampered enrolment [44] in Uganda and Rwanda respectively. Solidarity acts in community involves support from the community and social capital. One other outcome affecting uptake revealed by an included study was cultural belief (Nouna district in Burkina Faso) that setting money aside for health care could attract disease [41].

## Discussion

The studies included in this review originate from different disciplines including sociology, economics, and public health. Although there are some differences in terms of the methodology that each discipline applies, the included studies used either a purely qualitative approach, quantitative approach, or a mixed-method approach. The qualitative and quantitative analyses assessed different variables and were done in different countries. This led to contextual differences and variations in the interpretation and meaning of the results. The included studies differed considerably in study designs, settings and outcome measures; hence it was not possible to combine all results using meta-analyses. Thus, we use a narrative synthesis to present details for each study and discuss them in turn.

Age was found to be significantly related to uptake of the scheme; this review revealed that younger individuals were more willing to pay compared to older individuals. This finding was consistent across all included studies. Similarly, sex of the individual and household head is another significant determinant of enrolment. In countries like Burkina Faso and Nigeria, for instance, male headed households were found to be more likely to enrol as compared to female headed households [25, 26, 28, 30]. In the context of some African countries, this is not surprising as men are presumed to be responsible for financial decisions within the households.

This review identified education as playing a key role in uptake of CBHI and this finding was similar across all included studies; the less educated were willing to pay less compared to the more educated at both the household and individual levels. Furthermore, this review shows that the wealth or socioeconomic standing of households and individuals is associated with the willingness and ability to pay for health insurance [45–48]. This suggested that income or socioeconomic standing is very crucial in determining demand behaviour as found in the literature [49, 50]. However, for equity reasons, it is argued elsewhere that the use of health services should not be determined by ability to pay [1]. This is a crucial aspect that CBHI schemes need to pay attention to if there is a desire to cross-subsidise the poor. Usually these schemes charge a uniform premium and only those who can afford such premiums are able to pay to enrol.

Another key factor that affected uptake of the CBHI scheme was the household size. Included studies found that larger households were willing to pay higher amounts than relatively smaller households. However, this finding was not consistent across all included studies [51, 52]. Where larger households dropped out of the scheme this was likely a result of the huge financial burden faced by households when they seek health care; in many cases, the CBHI schemes are unable to cover the entire costs of health services. Membership of an already existing association in the community is also a determinant of enrolment. Households that were members of an association already were more willing to enrol into the scheme [22, 35]. Similarly, low-level community participation and involvement in the decision-making process [37, 38] and lack of solidarity acts in a community were found to hamper enrolment [44]. These reveal the role of solidarity and social cohesion in willingness to pay for the scheme [53]. These are very important elements in the design of health insurance. It is only through the acceptance of solidarity that individuals and households are willing to contribute towards the health care costs of others. Thus, building on social solidarity in designing CBHI schemes will increase acceptability and uptake.

In terms of health related variables, the quality of care was found to influence the uptake of the scheme. Individuals or households that perceived quality of care as good were more willing to pay than those who perceive the quality with less admiration [31, 39]. This is understandable within the context that people are less willing to pay for services generally that are of questionable quality. One study demonstrated the intersection of quality of health care and distance as households that perceived quality of health care centres in close proximity were willing to enrol into the scheme and pay a higher fee [29]. This would enable them to have access to other facilities that are farther away but with good quality of care.

In addition, household illness experiences also determined enrolment. Although in these studies no particular illness was identified [39], the results of some included studies revealed that households that have recorded sick members are less willing to pay than their counterparts. Perhaps this is as a result of an unpleasant experience. One of the empirical study included reported that households with more sick members were willing to pay more [28] which supports the notion that families with high illness rates or that are more prone to being ill have a greater tendency to participate or to be members of the health prepayment scheme [48]. On the other hand, fewer illness episodes in a specified period of time had a positive effect on drop-out of the scheme [27, 31]. The health status as recorded in some empirical studies also determined enrolment [27, 35, 39] as individuals with better health status in comparison with those with high illness rate (or poorer health status) were willing to pay lesser amounts for health insurance [23].

The use of modern medicine is also an important factor for enrolling into CBHI since the scheme requires the regular use of conventional means of treatment; hence those who use modern medicine have been found to be more willing to pay than those who use other means of treatment [22, 23, 36, 41]. This result points to the need to take preferences into account in designing any financing scheme. As the results from the review point out, households are more willing to pay if the CBHI provides the kind of services that they prefer.

Even though long distance to health facilities was not explicitly defined in the included studies, some of the studies report that traveling long distances to access health care makes households more willing to pay for CBHI [29, 32, 39]. This result diverges from some other studies that reported the opposite association between distances required to access healthcare and willingness to pay (or enrolment) [25, 27, 28]. In any case, it is inevitable that distance to health care is an important determinant of seeking health care. One of the issues that households will face in deciding to belong to the CBHI will be how to get to the facility closest to them. If transportation costs are not covered, this will have a huge impact on their willingness to pay particularly when the facility is far away from their place of residence.

The qualitative studies reiterated some crucial factors already highlighted in some of the quantitative studies and also pointed out some variables not measureable using quantitative methods. For instance, in most quantitative studies wealth was stated as a socio-demographic factor which has a profound impact on the uptake of the scheme. Also, as shown by quantitative studies, affordability is a key factor affecting enrolment. Non-enrolled individuals collectively identified a lack of financial means as the primary reason for not enrolling [37, 38, 41]. These results relating to affordability however it may be defined, present major challenges to the expansion of CBHI in many settings. One of these challenges is the regressivity of CBHI contributions [54]. Because a flat enrolment fee is charged, both the poor and the rich contribute the same amount in premium. From this premise, it is conceivable to find that the poor are unwilling to join the schemes. Part of this is the reason for the current debates around ensuring universal access to health services that many countries are buying into. However, these countries still struggle with covering those in the informal sector (especially the working poor), the vulnerable, the poor, and the unemployed. Because these groups of people are unable to afford payment for health services or to belong to the CBHI, there needs to be a way for others to contribute on their behalf. This is where the concept of solidarity discussed above becomes very relevant.

In terms of health related factors, perception of the quality of health care was also found to affect enrolment. Respondents criticised excessive prescribing, long waiting times, differential treatment, health provider’s attitude and technical incompetence amongst providers, irrespective of enrolment status [41–44]. These issues are those that may not be measured directly and could be subjective. As the results from the review indicate, there is a need to pay particular attention to them if a CBHI scheme is to attract more enrolees.

Related to these are some “software” characteristics. For instance trust, which was stressed in almost all the studies included [37, 38, 40, 41] is very relevant to enrolment. The quantitative studies revealed that household heads that have greater trust in the scheme were willing to pay higher amounts than their counterparts [29, 40]. Poor knowledge of the benefit package and poor understanding of the notion of the scheme were also found to affect enrolment [37, 38, 41]. Institutional rigidities in payment modality and timing of the enrolment campaign in relation to seasonal revenue fluctuations [43], were also found to contribute to the uptake of CBHI. One other outcome affecting uptake revealed by an included study was the cultural belief that setting money aside for health care could attract disease [41]. In such instance, people are unwilling to enrol into the scheme.

In summary, the main variables reiterated in both quantitative and qualitative studies as affecting enrolment included low levels of income or lack of financial resources, poor quality of health care services in terms of drug availability and medical supply, attitude of health care workers, patient waiting time and efficiency of treatment.

Furthermore, one important variable common in both types of studies was the importance of trust, in both the scheme and care providers. This is because the nature of the CBHI scheme is voluntary; therefore a level of trust is needed as it involves financial contribution from people. Low levels of trust in the insurance scheme can also be a result of previous negative experiences with insurance schemes. It is pertinent to note that, although we did not restrict study selection to a particular period, all studies included were done from 1990 onward. It could be inferred that this is the case since CBHI was not widely available before 1990 and the published literature only gained ground from this period onwards.

The use of a mixed-method approach [55–57] offers the opportunity for complementary answers to the research questions that could not be holistically answered by either qualitative or quantitative methods. This also generated a more relevant and robust review by maximising the findings and the ability of these findings to inform policy and practice. Thus, the fusion of both qualitative and quantitative evidence in this review enhanced its impact and effectiveness. Inclusion of both components can help identify priority research gaps and boost the relevance of the review for decision makers. The mixed methods also facilitated the incorporation of understanding of people’s diverse and contextual experiences from a qualitative perspective and robust quantitative estimates of benefits and harms.

The variety of studies included in the review provides a rich set of experiences that needs to be discussed in the context of the current debates around UHC. Internationally, it is argued that UHC cannot be achieved through voluntary means including community prepayment schemes. Also, evidence shows that CBHI schemes are unable to generate sufficient funds to cater for the health care needs of their catchment population. Enrolees are often entitled to a very limited benefit package which exposes them to out-of-pocket payments for services that are not covered. These together mean that promoting the widespread use of CBHI may counter the need to move towards UHC.

However, some communities, especially in Africa and Asia, have a large informal sector and a large rural community that makes it difficult to provide entire population coverage through government resources alone [58]. In some of these communities, one can argue that CBHI schemes may be relevant at least in the interim to provide some sort of coverage until there is a way to bring these schemes under a big umbrella. This type of approach has been used in countries like Ghana, Rwanda and Vietnam [58] with some degree of success. In Vietnam for instance, a voluntary scheme was introduced in 1994 that covers mainly informal workers and students. Gradually until 2008, the poor and the vulnerable were absorbed by an existing formal non-contributory scheme [59]. In Ghana, over 140 district wide CBHI schemes were formed and later integrated into the National Health Insurance Scheme [60]. In countries like Ghana and Rwanda, for instance, there are guidelines to exempt the poor and vulnerable from paying premiums and to provide subsidies to cover them under the national health insurance arrangement. However, there have been challenges with identifying the poor and vulnerable [60] and in many cases, there are no actuarial studies to determine the eventual cost of covering the poor and vulnerable using state resources.

Therefore, while in some cases CBHI schemes have proved helpful in the move to UHC, this may not always be the case as they present some challenges in terms of raising resources, proportion of the population covered (fragmentation), the benefit package, etc. Although voluntary prepayment schemes in themselves are not suited for achieving UHC, and there is no universally laid out path toward achieving UHC, countries that still use CBHI but aim to achieve UHC could build on this but ensure that the core principles of equity, fairness, sustainability and efficiency are met. More importantly, whatever form of arrangements are in place, they should guarantee the population access to quality health care that is affordable for which they do not have to suffer any financial hardships in using them (i.e., the core of UHC) [61].

### Limitations

For some variables or characteristics, variations were reported in the different countries. Some variables were positively significant while others were negatively significant. However, this could be linked to contextual differences in these countries. Thus, it makes it difficult to reach a conclusion with regards to the impact of each variable on enrolment. Because most included quantitative studies were cross-sectional, the study design has a basic limitation in assessing the direction of ‘causality’ between the outcome and exposure. Only relationships and associations can be deduced.

Another limitation of this review is that only studies conducted in English were included. Some other studies that may meet the inclusion criteria but were written in other languages were excluded. Also, the role of non-governmental organisations and the public health system were not considered as none of the included studies considered this key area.

## Conclusion

The review has pointed out some important aspects relating to enrolment in CBHI. In the current debate about ensuring UHC, although there are arguments against voluntary schemes, CBHI schemes, where they currently exist, may still serve as a means to providing health insurance to those in the informal sector as well as those in rural locations. However, it needs to address some issues relating to lack of funds, poor quality of care, and lack of trust which are major reasons for low willingness to enrol in CBHI in LMICs. Thus, if CBHI schemes are to serve as a means to providing access to health services, at least in the short term, then attention should be paid to the issues that militate against their success.

## Additional files

Additional file 1: Table S1. PUBMED search strategy. (DOCX 12 kb) Additional file 2: Table S2. Excluded studies [34, 62–71]. (DOCX 12 kb) Additional file 3: Table S3. Summary findings from included studies [19–33, 35–44]. (DOCX 17 kb)
