# Supplementary material for: A systematic review of factors that affect uptake of community-based health insurance in low-income and middle-income countries
Source: BMC Health Serv Res. 2015 Dec 8;15:543. doi: 10.1186/s12913-015-1179-3 (PMC4673712; doi:10.1186/s12913-015-1179-3)
Supplement: Additional file 1: Table S1. — PUBMED search strategy. (DOCX 12 kb) [file 12913_2015_1179_MOESM1_ESM.docx]

**Additional file 1: Table S1: PUBMED search strategy**

|  | **Query** |
| --- | --- |
| #1 | "health insurance"[MeSH Terms] |
| #2 | “community based” OR “rural” OR “mutual” OR “micro” OR “community” OR “group” |
| #3 | #1 AND #2 |
| #4 | (Afghanistan OR Islamic Republic of Afghanistan OR Bangladesh OR People's Republic of Bangladesh OR Benin OR Dahomey OR Republic of Benin OR Burkina Faso OR Burkina OR Republic of Upper Volta OR Burundi OR Republic of Burundi OR Cambodia OR Kingdom of Cambodia OR Central African Republic OR Chad OR Republic of Chad OR Comoros OR Union of the Comoros OR Democratic Republic of the Congo OR DR Congo OR Congo-Kinshasa OR DRC OR Zaire OR Eritrea OR State of Eritrea OR Ethiopia OR Federal Democratic Republic of Ethiopia OR The Gambia OR Republic of the Gambia OR Guinea OR Republic of Guinea OR Guinea-Conakry OR Guinea-Bissau OR Republic of Guinea-Bissau OR Haiti OR Republic of Haiti OR Kenya OR Republic of Kenya OR North Korea OR Democratic People's Republic of Korea OR Kyrgyz Republic OR Kyrgyzstan OR Liberia OR Republic of Liberia OR Madagascar OR Republic of Madagascar OR Malawi OR Republic of Malawi OR The Warm Heart of Africa OR Mali OR Republic of Mali OR Mozambique OR Republic of Mozambique OR Myanmar OR Burma OR Republic of the Union of Myanmar OR Nepal OR Democratic Republic of Nepal OR Niger OR Republic of Niger OR Rwanda OR Republic of Rwanda OR Sierra Leone OR Republic of Sierra Leone OR Somalia OR Federal Republic of Somalia OR South Sudan OR Republic of South Sudan OR Tajikistan OR Republic of Tajikistan OR Tanzania OR United Republic of Tanzania OR Republic of Tanganyika and Zanzibar OR Togo OR Togolese Republic OR Uganda OR Republic of Uganda OR Zimbabwe OR Republic of Zimbabwe OR Rhodesia) |
| #5 | (Armenia OR armenia OR Bhutan OR Kingdom of Bhutan OR Bolivia OR Plurinational State of Bolivia OR Cameroon OR Republic of Cameroon OR Republic of Cameroun OR Cape Verde OR Republic of Cape Verde OR Cote D'ivoire OR Ivory Coast OR Republic of Cote D'ivoire OR Djibouti OR Republic of Djibouti OR Arab Republic of Egypt OR Egypt OR El Salvador OR Georgia OR Ghana OR Republic of Ghana OR Guatemala OR Republic of Guatemala OR Guyana OR Co-operative Republic of Guyana OR Honduras OR Republic of Honduras OR Spanish Honduras OR Republic of Indonesia OR Indonesia OR India OR Republic of India OR Kiribati OR Republic of Kiribati OR Kosovo OR Kosovo and Metohija OR Laos OR Lao Lao People's Democratic Republic OR Lesotho OR Kingdom of Lesotho OR Mauritania OR Islamic Republic of Mauritania OR Micronesia, Fed. Sts. OR Federated States of Micronesia OR FSM OR Moldova OR Republic of Moldova OR Mongolia OR Morocco OR Kingdom of Morocco OR Nicaragua OR Republic of Nicaragua OR Nigeria OR Federal Republic of Nigeria OR Pakistan OR Islamic Republic of Pakistan OR Papua New Guinea OR Independent State of Papua New Guinea OR Paraguay OR Republic of Paraguay OR Philippines OR Republic of the Philippines OR Samoa OR Independent State of Samoa OR Sao Tome and Principe OR Democratic Republic of Sao Tome and Principe OR Senegal OR Republic of Senegal OR Solomon Islands OR Sri Lanka OR Democratic Socialist Republic of Sri Lanka OR Sudan OR Republic of the Sudan OR North Sudan OR Swaziland OR Kingdom of Swaziland OR Ngwane OR Yuwatini OR Syrian Arab Republic OR Syria OR East Timor OR Timor-Leste OR Democratic Republic of Timor-Leste OR Ukraine OR Uzbekistan OR Republic of Uzbekistan OR Vanuatu OR Republic of Vanuatu OR Vietnam OR the Socialist Republic of Vietnam OR West Bank and Gaza OR Yemen OR Yemeni Republic OR Zambia OR Republic of Zambia.) |
| #6 | (Angola OR Republic of Angola OR Albania OR Republic of Albania OR Algeria OR The People's Democratic Republic of Algeria OR American Samoa OR Argentina OR Azerbaijan OR Belarus OR Belize OR Bosnia and Herzegovina OR Bosnia-Herzegovina OR Bosnia OR Botswana OR Brazil OR Federative Republic of Brazil OR Bulgaria OR China OR People's Republic of China OR Colombia OR Costa Rica OR Fiji OR Gabon OR Gabonese Republic OR Grenada OR Hungary OR Islamic Republic of Iran OR Persia OR Iran OR Iraq OR Jamaica OR Jordan OR Hashemite Kingdom of Jordan OR Kazakhstan OR Lebanon OR Lebanese Republic OR Libya OR State of Libya OR Macedonia OR Republic of Macedonia OR Malaysia OR Maldives OR Republic of the Maldives OR Maldives Islands OR Marshall Islands OR Republic of the Marshall Islands OR Palau OR Republic of Palau OR Panama OR Republic of Panama OR Peru OR Romania OR Serbia, OR the Republic of Serbia OR Seychelles OR the Republic of Seychelles OR South Africa OR Saint Lucia OR Saint Vincent and the Grenadines OR Suriname OR Thailand OR Kingdom of Thailand OR Tonga OR Kingdom of Tonga OR Tunisia OR Turkey OR Turkmenistan OR Turkmenia OR Cuba OR Dominica OR Commonwealth of Dominica OR The Dominican Republic OR Ecuador OR Mauritius OR Mexico OR United Mexican States OR Montenegro OR Namibia OR Tuvalu OR Ellice Islands OR Venezuela OR the Bolivarian Republic of Venezuela) |
| #7 | (Low-income country OR lower-income country OR third-world country OR middle-income country) |
| #8 | developing countries[MeSH Terms] |
| #9 | #4 OR #5 OR #6 OR #7 OR #8 |
| #10 | #3 AND #9 |
