# Supplementary material for: A systematic review of factors that affect uptake of community-based health insurance in low-income and middle-income countries
Source: BMC Health Serv Res. 2015 Dec 8;15:543. doi: 10.1186/s12913-015-1179-3 (PMC4673712; doi:10.1186/s12913-015-1179-3)
Supplement: Additional file 2: Table S2. — Excluded studies [34, 62–71]. (DOCX 12 kb) [file 12913_2015_1179_MOESM2_ESM.docx]

**Additional file 2: Table S2: Excluded studies**

| Author | Year | Country | Reason for Exclusion |
| --- | --- | --- | --- |
| Asgary et al.[34] | 2004 | Iran | This study focused on health insurance (which could be any type of health insurance) not community-based health insurance specifically |
| Eckhardt et al.[62] | 2011 | Ecuador | This is a feasibility study and also identifying people’s understanding and attitudes toward the presented CBHI model. |
| Dong et al.[63] | 2004 | Burkina Faso | This is also a feasibility study and willingness to pay was considered in terms of amount willing to pay and not factors that affect the willingness to pay |
| Criel et al.[64] | 1998 | Democratic Republic of Congo | The paper investigates social perceptions of the Bwamanda health insurance scheme, hence factors affecting enrolment is implicit in the study. |
| Barnighausen et al.[65]^58^ | 2007 | China | Considers informal sector workers’ willingness to pay for social health insurance. |
| Katajima[66] | 1999 | Tokyo | Study was conducted in Tokyo, a high-income country. |
| Lofgren [67] | 2008 | Vietnam | The study explored both mandatory and voluntary insurance broadly whereby voluntary insurance could be private health insurance or community-based health insurance, not specified. |
| Taylor et al.[68] | 2006 | America | The study looked at community-based programmes in the United States a high income country. |
| Dong et al.[69] | 2003 | Burkina Faso | A comparison of the reliability of the take-it-or-leave-it and the bidding game approach to determine willingness to pay is the focus of this study. The study assessed the agreement between test and retest. |
| Walraven[70] | 1996 | Tanzania | Willingness to pay for district hospital services in a rural area, which is out-of-pocket payment at the point of health care. |
| Vellakkal[71] | 2013 | India | Determinant of enrolment in voluntary health insurance was considered not community-based health insurance specifically. |
