# Supplementary material for: A systematic review of factors that affect uptake of community-based health insurance in low-income and middle-income countries
Source: BMC Health Serv Res. 2015 Dec 8;15:543. doi: 10.1186/s12913-015-1179-3 (PMC4673712; doi:10.1186/s12913-015-1179-3)
Supplement: Additional file 3: Table S3. — Summary findings from included studies [19–33, 35–44]. (DOCX 17 kb) [file 12913_2015_1179_MOESM3_ESM.docx]

**Additional file 3: Table S3: Summary findings from included studies**

| **Study ID** | **Findings** |
| --- | --- |
| **Quantitative studies** | |
| Ataguba 2008[39] | Male-headed households are more willing to pay than female-headed households; wealthy households are also more willing to pay than less wealthy households.  Household heads that are less educated are willing to pay lesser amounts than those who are highly educated. This bears correlation with the level of income because the non-poor are more likely to be more educated that the poor.  Households that have to travel long distances to access health care are more willing to pay than households that have to travel less distances.  Households that perceive the quality of health centre services as good are more willing to pay than those that perceive the quality as lesser.  Households that have recorded a household member sick are less willing to pay than their other counterparts  Respondents with better health status are willing to pay lesser amounts when compared with respondents with poorer health status. |
| Ataguba 2008[29] | Wealthier households are willing to pay higher amounts than households that are less wealthy.  Male headed households are willing to pay higher amounts than female headed households.  Households with larger sizes are also willing to pay higher amounts than households with relatively smaller size.  Household that perceives the quality of health care centres nearest to them as poor are willing to pay more than households that perceive the quality to be good. Household heads that have greater trust and confidence in the proposed scheme are more willing to pay higher amounts than those that have low confidence in the scheme.  Households that have to travel long distances to access health care are willing to pay higher amounts than their counterparts that live close to health care centres. |
| Banwat[19] | Age was statistically significant with willingness to pay.  Willingness to pay was highest amongst the tertiary level education  Statistically significant association was reported between marital status and willingness to pay |
| Binnendijk 2013[20] | Average willingness to pay for health insurance varies greatly across the locations.  Willingness to pay increases as the income of the household increases and there is also great variation of willingness to pay across income groups within the locations. |
| Chankova 2008[21] | Female household heads are more likely to join mutual health organisation (MHO)  Education of head of household is also positively associated with MHO enrolment in all three settings  Older age of the household head is significantly associated with enrolment only in Ghana and Senegal.  Household economic status indicates that individuals from the richest quintile are more likely to be enrolled compared with those from poorest quintile.  Availability of a health facility in the community is associated with higher likelihood of enrolment in Mali and Senegal. |
| Donfouet 2011[23] | Age is statistically significant, younger individuals are more willing to pay than older ones  Farmers are less willing to pay than those who are self-employed or private/public sector  Income has a positive significant impact on willingness to pay. Health status does not affect the decision of the respondents to pay for community prepayment scheme.  Catholic household heads are more willing to pay than those from other religion  Households more knowledgeable about CBHI tend to be more willing to pay  Those who seek orthodox medicine are more willing to pay than those who use other means |
| Donfouet 2013[22] | Household heads who have been more involved in associations are more willing to pay for CBHI.  Household from catholic religion has a higher willingness to pay than those practicing other religions, or not practicing any religion.  Household heads that regularly use clinics/hospitals when sick are more willing to pay than those who use traditional healers and herbalists. |
| Dong 2002[26] | The aged are more willing to pay less than the young  Married men are more willingness to pay more than married women  Education influenced male willingness to pay not female  Male heads of household were willing to pay more than female heads; other males were willing to pay more than other females also.  Distance to health facility significantly influence both male and female willingness to pay |
| Dong 2004[25] | Household heads residing in Nouna town were willing to pay relatively less for themselves and more for other household members than those in rural areas. As the household size increases the willingness to pay difference increases.  Greater distance to the health facility had the expected negative association, reducing individual willingness to pay.  The old have a lower willingness to pay than the young, females have lower willingness to pay than males, the poor have a lower willingness to pay than the rich, and those with less schooling have a lower willingness to pay than those with more years of schooling. |
| Dong 2009[31] | The following factors all had a positive effect on drop-out, meaning that they increased the probability that a household did not renew its membership in CBI: female household head, higher household head’s age, lower household head’s education, larger household size, living in rural area, lower number of illness episodes in the past three months, fewer children or elderly in a household, poor perceived quality of care, less health care seeking in the month prior to the survey. |
| Dror 2007[27] | High significant positive association between willingness to pay and household size  Significant association between education of the household head and willingness to pay.  Marginally significant association between age of household head and willingness to pay.  A significantly high and strong positive association with household income. There is a marginally significant negative correlation between distance to hospital and willingness to pay.  Households who experienced hospitalisation in the last 2 years were willing to pay more |
| Dong 2003[24] | Living in Nouna town reduce the value of willingness to pay. However age, sex, education, monogamy, traditional religion increases the value of willingness to pay. |
| Mathiyazhagan 1998[28] | Family size of households strongly influenced willingness to pay for rural health insurance.  There is a negative relationship between age and willingness to pay for the scheme.  Higher income level households have a higher chance of willingness to pay for the proposed scheme.  The higher the number of days lost to ill-health, the more likely someone is to pay for the scheme.  Distance, travel and waiting time to obtain health care were used as proxies for the physical accessibility of the respondents. The estimated coefficients of physical accessibility are significantly positive in all cases except one variable (i.e. waiting time). |
| Onwujekwe 2009[30] | Willingness to pay was positively related to socio-economic status for both respondents and other household members. Conversely, willingness to pay was negatively related to geographic location, showing that residence in rural areas led to decreased willingness to pay.  Positive relationship of gender with willingness to pay, implying higher willingness to pay amongst males compared with females.  Additionally, the higher the number of years of education, the higher were the amounts of elicited willingness to pay. |
| Oriakhi 2012[36] | Household with more members are more likely to participate in the scheme  Respondents with high income are less likely to participate  Households with higher medical expenses and medical credit are more likely to enrol |
| Allegri 2006[32] | In comparison with controls, cases were more likely to belong to the Bwaba ethnic group, employed outside the farming sector, and were more likely to fall into the wealthier categories.  Positive association between enrolment in CHI and higher education, higher socioeconomic status, a negative perception of the adequacy of traditional care, a higher proportion of children living within the household, an increased distance to the health facility, and a lower level of socioeconomic inequality within the community.  Cases also differed from controls in that they were more likely to have rated traditional care as “mediocre/inadequate”, to have used curative health services, to live far from the health facility, and to live in communities with low levels of socioeconomic inequality. |
| Shafie 2013[33] | Ethnicity, marital status and higher education attainment showed significant association for willingness to pay  Uptake is positively affected by a higher income  Chinese ethnic group are more willing to enrol in the scheme.  Willingness to pay is higher for those that have current health insurance, higher incomes and people of Chinese ethnicity but decreases for those who are married |
| Ozawa 2009[40] | Villagers who renewed insurance (RRR = 1.15) scheme were found to have statistically significantly higher trust levels compared to those who are new to the scheme (RRR = 1.08) and those who dropped out (RRR = 1.06) |
| Binam 2004[35] | Household revenue level, gender, constant visit to the health centre, household association experience and the household health status affect significantly the willingness to prepay value.  A positive and significant membership to a community club or association increases willingness to prepay for the scheme.  Positive and statistically robust relationship between the family health status and enrolment.  Respondent’s monthly revenue and frequent use of health facility have a positive and statistically significance with the willingness to pay |
| Qualitative studies | |
| De Allegri 2006[41] | Non-enrolled individuals unanimously identified lack of means as the primary reason for not joining the scheme.  Once probed, respondents expressed dissatisfaction with the quality of care. Respondents, irrespective of enrolment status, criticised the quality of care. Criticisms always focused on three aspects: long waiting times, excessive prescribing, and differential treatment depending on the patient’s socio-economic status.  Interviewees were reluctant to justify low enrolment in relation to cultural beliefs and practices. All interviewees acknowledged the fact that setting money aside for health care may be perceived as attracting diseases, but they always attributed this belief to others in their community.  All enrolled individuals constructed a link between their choice to enrol and the trust they have in the CBI management. Interviewees explained scepticism in relation to three factors: lack of adequate knowledge and understanding, health providers’ resistance towards the new initiative, and previous bad experiences with collective arrangements. |
| Basaza 2007[37] | Incapability to raise the contributions  Lack of trust in local financial organisations after previous depressing experiences with similar institutions  Low level of community involvement in the management of hospital-based CHI schemes  Lack of information on and poor understanding of the notion of community health insurance |
| Allegri 2005[43] | Institutional rigidity in payment modality was identified to be a greater barrier to enrolment.  Respondents also asked that the timing of the enrolment campaign be changed to better match seasonal revenue fluctuations.  Knowledge of the benefit package was very poor among most respondents in rural as well as in urban settings.  Most interviewees, regardless of socio-economic and insurance status, could not recall the specific elements of the package  Respondent’s defined poor quality of care exclusively in terms of health providers’ attitudes, what they expect from the scheme is not to seek an alternative contracting partner, but rather to invest in changing providers’ attitudes and induce the quality improvements. |
| Basaza 2008[38] | There is mixed understanding of pooling of contributions in the scheme.  The level of trust in financial organisations stands out clearly as a key factor affecting enrolment in the scheme.  Inability to pay for membership was pointed out as the foremost reason for not joining the schemes.  The desire to have members more closely involved in the decision-making process on the scheme; and the wish to include chronic diseases in the package of benefits. |
| Criel 2003[42] | Quality of care is perceived as unsatisfactory; hence people are not motivated to join the scheme. |
| Ozawa 2009[40] | The need for health insurance organisation to build trust via having interpersonal relationships with villagers. |
| Schneider 2005[44] | People’s mistrust in provider is the main reason for low enrolment.  Technical incompetence amongst provider is also reason for low enrolment.  In areas where there are some solidarity acts, high enrolment occurred there. |
